# Aberrant transcriptional and post-transcriptional regulation of SPAG5, a YAP-TAZ-TEAD downstream effector, fuels breast cancer cell proliferation

**DOI:** 10.1038/s41418-020-00677-9

**Published:** 2020-11-23

**Authors:** Valeria Canu, Sara Donzelli, Andrea Sacconi, Federica Lo Sardo, Claudio Pulito, Noa Bossel, Anna Di Benedetto, Paola Muti, Claudio Botti, Eytan Domany, Silvio Bicciato, Sabrina Strano, Yosef Yarden, Giovanni Blandino

**Affiliations:** 1 Oncogenomic and Epigenetic Unit, Department of Research, Diagnosis and Innovative Technologies, IRCCS Regina Elena National Cancer Institute, Rome, Italy; 2 Clinical Trial Center, Biostatistics and Bioinformatics Unit, IRCCS Regina Elena National Cancer Institute, Rome, Italy; 3 Department of Physics of Complex Systems, Weizmann Institute of Science, Rehovot, 7610001 Israel; 4 Department of Pathology, IRCCS Regina Elena National Cancer Institute, Rome, Italy; 5 Department of Biomedical Science and Oral Health, University of Milan, Milan, 20122 Italy; 6 Breast Surgery Unit, IRCCS Regina Elena National Cancer Institute, Rome, Italy; 7 Center for Genome Research, Department of Biomedical Sciences, University of Modena and Reggio Emilia, Modena, Italy; 8 SAFU Unit, Department of Research, Diagnosis and Innovative Technologies, IRCCS Regina Elena National Cancer Institute, Rome, Italy; 9 Department of Biological Regulation, Weizmann Institute of Science, Rehovot, 7610001 Israel

**Keywords:** Tumour biomarkers, Diagnostic markers

## Abstract

Sperm-associated antigen 5 (SPAG5) is an important driver of the cell mitotic spindle required for chromosome segregation and progression into anaphase. SPAG5 has been identified as an important proliferation marker and chemotherapy-sensitivity predictor, especially in estrogen receptor-negative breast cancer subtypes. Here, we report that SPAG5 is a direct target of miR-10b-3p, and its aberrantly high expression associates with poor disease-free survival in two large cohorts of breast cancer patients. SPAG5 depletion strongly impaired cancer cell cycle progression, proliferation, and migration. Interestingly, high expression of SPAG5 pairs with a YAP/TAZ-activated signature in breast cancer patients. Reassuringly, the depletion of YAP, TAZ, and TEAD strongly reduced SPAG5 expression and diminished its oncogenic effects. YAP, TAZ coactivators, and TEAD transcription factors are key components of the Hippo signaling pathway involved in tumor initiation, progression, and metastasis. Furthermore, we report that SPAG5 is a direct transcriptional target of TEAD/YAP/TAZ, and pharmacological targeting of YAP and TAZ severely reduces SPAG5 expression. Collectively, our data uncover an oncogenic feedback loop, comprising miR-10b-3p, SPAG5, and YAP/TAZ/TEAD, which fuels the aberrant proliferation of breast cancer.

## Introduction

Breast cancer is the most frequent type of tumor and the main cause of cancer-related death among women worldwide [1]. Based on the expression of estrogen receptor (ER), progesterone receptor (PgR), and ERBB2 (HER2), breast tumors are classified as luminal A, luminal B, HER2, and basal-like or triple-negative (TNBC), which exhibit different tumor aggressiveness and response to therapy [2–4]. It is well established that aberrant expression of miRNAs, a class of short noncoding endogenous RNAs that regulate gene expression by binding to the 3′-untranslated regions of their target mRNAs, fosters cancer development [5]. Several miRNAs have been reported to act as regulators of breast cancer development and progression and correlate with the hormone receptor status [6].

Sperm-associated antigen 5 (SPAG5), primarily described as a component of the mitotic spindle required for entry into anaphase [7–12], acts as a driver oncogene in various cancer types [13–17], including triple-negative breast cancer [18–20].

Advanced breast tumors frequently display increased activity of the Hippo signaling pathway [21–23], whose key players are the TEAD-transcriptional coactivator Yes-associated protein (YAP) and the transcriptional coactivator with a PDZ-binding domain (TAZ) [24]. Here, we report for the first time that SPAG5 serves as a direct target of miR-10b-3p. Accordingly, miR-10b-3p’s expression is significantly anticorrelated to SPAG5’s transcript level. The latter associates with poor survival in two different cohorts of breast cancer patients, METABRIC and TCGA datasets. Congruently, SPAG5 depletion reduces proliferation and migration of breast cancer cell lines, while its ectopic overexpression confers oncogenic properties to MCF-10A, an untransformed mammary gland cell line. Importantly, high expression of SPAG5 is linked to the transcriptional signature of activated YAP/TAZ in breast cancer patients, implying the involvement of YAP/TAZ in the oncogenic activity of SPAG5. We further report that SPAG5 is a direct transcriptional target of the YAP/TAZ/TEAD axis, such that YAP/TAZ depletion diminishes SPAG5’s oncogenic potential. Gratifyingly, pharmacological inactivation of YAP and TAZ markedly reduced SPAG5 expression. In aggregate, our findings uncover a transcriptional and post-transcriptional network that sustains aberrant SPAG5 expression and contributes to breast cancer aggressiveness.

## Materials and methods

### CoSMic analysis using the METABRIC dataset

The METABRIC dataset contains 1286 primary breast cancer tumors for which both mRNA https://ega-archive.org/dacs/EGAC00001000484 [25] and miR https://ega-archive.org/studies/EGAS00000000122 [26] expression data were measured. The patients are from the 5 subtypes of breast cancer: Her2 + (127 pt.), basal-like (209 pt.), luminal A (479 pt.), luminal B (312 pt.), and normal-like (151 pt.).

We used CoSMic along with the METABRIC dataset and miRanda predictions (http://www.ebi.ac.uk/enright-srv/microcosm/htdocs/targets/v5/) [27].

CoSMic algorithm integrates gene expression measurements of mRNAs and miRs taken from the same samples, with sequence-based miR-target predictions, to improve the reliability of miR-target predictions. The algorithm works on each miR separately, and searches for a group of genes with expression levels that are significantly anticorrelated with the miR’s expression, and have high sequence-based prediction scores. CoSMic provides for each miR a group of genes that were identified as its predicted context-specific targets and a corresponding *P* value for the significance of this prediction.

### Statistical analysis

Normalized miRNA and gene expression of breast cancer patients was obtained from Metabric dataset and Broad Institute TCGA Genome Data Analysis Center (2016): TCGA data from Broad GDAC Firehose 2016_01_28 run. Broad Institute of MIT and Harvard. Dataset. 10.7908/C11G0KM9.

Statistical significance of the modulation of a gene among subgroups of patients was inferred by Student’s *T* test or ANOVA test.

Unsupervised hierarchical clustering was performed to individuate a specific pattern of expression using the Euclidean distance metric.

Survival and progression-free survival were evaluated by Kaplan–Meier method and a log-rank test was used to establish the statistical significance of the distance between curves. High- and low- expression values for subgroups of patients were assessed by calculating *z* scores of a single gene or by the *z* scores of the mean value of a signature of genes. The multivariate Cox proportional hazard regression model was used to evaluate the impact of clinical variables on the survival curves. MATLAB R2019b software was used to perform the analyses.

### Collection of breast cancer gene expression data

To investigate the correlation between SPAG5 expression and metastasis-free survival, we used a collection of 3661 unique samples from 25 independent cohorts comprising gene expression data of breast cancer samples annotated with histological tumor grade and clinical outcome. The collection was normalized and annotated with clinical information as described in ref. [28].

### Survival analysis

To identify two groups of tumors with either high or low SPAG5 expression, we used the classifier described in ref. [29]. Tumors were classified as “SPAG5 Low” if the SPAG5-standardized expression was negative and as “SPAG5 High” vice versa. To identify two groups of tumors with either high or low YAP/TAZ signature, we used the same classification rule on the YAP/TAZ signature form [30]. Tumors were classified as “YAP/TAZ signature Low” if the signature score was negative and as “YAP/TAZ signature High” if the signature score was positive. To evaluate the prognostic value of SPAG5 expression, we estimated, using the Kaplan–Meier method, the probabilities that patients would remain free of metastasis. The Kaplan–Meier curves were compared using the log-rank (Mantel–Cox) test. *P* values were calculated according to the standard normal asymptotic distribution. Survival analysis was performed in GraphPad Prism.

### Signature scores

Signature scores have been obtained summarizing the standardized expression levels of signature genes into a combined score with zero means [29]. The values shown in graphs are thus adimensional.

### Cell lines and culture conditions

Human cell lines MDA-MB-231, MDA-MB-468, and MCF-7 were maintained in Dulbecco’s Modified Eagle Medium DMEM (Invitrogen–GIBCO, Carlsbad, CA, USA) supplemented with 10% fetal bovine serum (FBS) (Invitrogen–GIBCO), penicillin (100 U/ml), and streptomycin (100 µg/ml). SUM-159PT human cell line was maintained as DMEM/F12.GLUTAMAX (Invitrogen–GIBCO, Carlsbad, CA, USA) supplemented with 10% fetal bovine serum (FBS) (Invitrogen–GIBCO), 10 µg/ml Insulin (Sigma, St Louis, MO, USA), penicillin (100 U/ml), and streptomycin (100 µg/ml). MCF-10A human cell line was maintained as DMEM/F12.GLUTAMAX (Invitrogen–GIBCO, Carlsbad, CA, USA) supplemented with 5% horse serum (HS) (Invitrogen–GIBCO), 10 µg/ml Insulin (Sigma, St Louis, MO, USA), 20 ng/ml EGF, 0.5 µg/ml hydrocortisone, penicillin (100 U/ml), and streptomycin (100 µg/ml). All cell lines were obtained from ATCC and cultured as monolayers at 37 C and 5% CO_2_.

### Immunohistochemistry

Ten breast cancer and the correspondent nontumoral tissues were randomly selected from 80 cases already evaluated for miRNA profiles. The study was approved by the scientific ethics committee from Regina Elena Cancer Institute (protocol number 5E/459/10).

Formalin-fixed paraffin-embedded BC specimens were cut on SuperFrost Plus slides (Menzel-Glaser, Braunschweig, Germany). Two-micron-thick paraffin-embedded sections were stained with a streptavidin-enhanced immunoperoxidase technique (Supersensitive Multilink, Novocastra, Menarini Florence, Italy) in an automated autostainer (Bond Max, Menarini) using the following reagents: anti-SPAG5 polyclonal antibody (PoAb) 1:200, Bethyl Laboratories, Inc. (Montgomery, TX). The pH 8 buffer antigen-retrieval protocols were applied. Diaminobenzidine (Menarini) was used as a chromogenic substrate.

The immunostaining was considered positive when at least 10% of the neoplastic cells showed a distinct cytoplasmic and/or nuclear/cytoplasmic immunoreactivity. Two investigators performed an evaluation of the immunohistochemical results, blinded to all patient data, independently. *H* score = [1× (% cells 1 + ) + 2× (% cells 2 + ) + 3× (% cells 3 + )].

### Immunocytochemistry and immunofluorescence

For immunocytochemistry assay, cells were seeded onto glass coverslips (Paul Marienfeld, Lauda-Königshofen, Germany) in six-well dishes (Corning Inc.) at 4 × 10^4^ cells/well in culture media, and fixed with 4% formaldehyde in PBS for 15 min at room temperature. Cells were permeabilized with 0.25% Triton X-100 in PBS for 10 min. After washing with PBS, the coverslips were incubated with peroxidase inhibitor and then with anti-SPAG5 antibody A301-512A Bethyl Laboratories, Inc. (Montgomery, TX) diluted in 5% bovine serum albumin (BSA)/PBS for 2 h at room temperature. Protein staining was revealed through DAB enzymatic reaction, and nuclei were counterstained with hematoxylin.

For immunofluorescence, cells were fixed and permeabilized as already described. Slides were blocked for 30 min in 5% BSA/PBS at room temperature and then incubated with an anti-SPAG5 antibody A301-512A Bethyl Laboratories (Montgomery, USA), anti-γ-tubulin sc-17787 (Santa Cruz), according to the manufacturer’s instructions, in 5% BSA/PBS overnight at +4 °C. Cells were incubated with secondary antibody Alexa Fluor 594 (1:500, Thermo Fisher Scientific) for 45 min. Nuclei were stained with DAPI (Thermo Fisher Scientific).

### Plasmids and transfections

For mature miR-10b-3p expression, we used Pre-miRNA Precursor-Negative Control (Ambion), Pre-miRNA10b-3p (Ambion), miRNA inhibitor negative control (Ambion), and miRNA-10b-3p inhibitor (Ambion) at a final concentration of 5 nM using *Lipofectamine RNAi* MAX (Invitrogen) according to the manufacturer’s instructions. MDA-MB-231, MDA-MB-468 SUM-159PT MCF-7, and MCF-10A cells were transfected with siRNA of SPAG5: siSPAG5 #1 AAGGAGUCUGAAACAGAAGAU siSPAG5 #2 AAGGCAGCAACAACUCAUCUC, siYAP GACAUCUUCUGGUCAGAGA, siTAZ is a pool of two independent siRNAs mixed in an equal amount of siTAZ#1 AAAGUUCCUAAGUCAACGU, and siTAZ#2 AGGUACUUCCUCAAUCACA and siTEAD GCAUUUGUAUACCGAAUAA at a final concentration of 100 pM using *Lipofectamine RNAi* MAX (Invitrogen) according to the manufacture’s instructions. siGFP UUCAGC GUGUCCGGGGAG was used as control. For stable overexpression of SPAG5, MCF-10A cells were transfected with a plasmid pcMV6, containing the complete open-reading frame of the human SPAG5 transcript driven by the CMV promoter (OriGene Technologies) using *Lipofectamine 3000* (Invitrogen) according to the manufacturer’s instructions. No difference between the efficiency of the transfection of empty vector pcMV6 and pvMV6-SPAG5 plasmid was assessed. Culture media containing G-418 A1720 (Sigma-Aldrich) at final concentration of 0.1 µM, was used to select individual clones that were screened for SPAG5 overexpression by western blot analysis. After 3 months of continuous culture in selection media containing G-418, SPAG5 overexpression was unchanged.

### FACS cell cycle analysis

For cell cycle analysis, cells were collected after 72 h from selective depletion. Fixed cells were treated for 30 min with RNase before PI addiction and analyzed with Guava Easycyte 8HT flow cytometer equipped with Guava Soft 2.1 (Millipore) according to the manufacturer’s instructions.

### BrdU-incorporation analysis

Cells were grown in complete medium with 10 µM of BrdU for 4 h, washed with BSA/PBS, and fixed. After denaturation, cells were treated with anti-BrdU primary antibody. Then, permeabilized cells were labeled with fluorescent secondary antibody and treated with propidium iodide before FACS analysis according to the manufacturer’s instructions.

### Annexin V staining

Annexin staining was performed following Annexin V-FITC Apoptosis Detection Kit protocol (eBioscence) [31] according to the manufacturer’s instructions.

### Luciferase assay

pMirTarget vector containing full length of 3′-UTR of the human SPAG5 gene was purchased from OriGene (OriGene Technologies).

SPAG5 3′UTR mutant was made with the QuikChange site-directed mutagenesis kit (Stratagene) using the following primers:

SPAG5 3′UTR del 174–176 FW: GAGCTAAGAAACTGAAAGCCAGATGCTTCACCTCT;

RV: AGAGGTGAAGCATCTGGCTTTCAGTTTCTTAGCTC.

HEK-293 cells were transfected using Lipofectamine 2000 (Invitrogen) with pMiR-Target vector containing the 3′-UTR of SPAG5, together with Pre-miRNA Precursor-Negative Control (Ambion) and Pre-miRNA10b-3p (Ambion) or pMiR-Target vector from OriGene (OriGene) with Pre-miRNA Precursor-Negative Control (Ambion) and Pre-miRNA10b-3p (Ambion) in 24-well plates. Firefly and Renilla luciferase activities were measured 48 h post transfection using the Dual-Luciferase Reporter Assay System (Promega) in the GloMax 96 Microplate Luminometer (Promega). Renilla luciferase was used to normalize the firefly luciferase.

pGL3-SPAG5 657–884 and pGL3-SPAG5 2909–3084 were generated by cloning 227 bp and 175 bp, respectively, from SPAG5 promoter in the SmaI- and XhoI-restriction sites of pGL3-basic vector (Promega) using the following primers:

pGL3-SPAG5 657–884 FW: ATTTCCCGGGGCACGGACTCTTTCATCCA;

pGL3-SPAG5 657–884 RV: ATCTCTCGAGGCCTCCCAAAGTGCTAGGAT;

pGL3-SPAG5 2909–3084 FW: ATTTCCCGGGTGGGGTCGTTCATGACTGTA;

pGL3-SPAG5 2909–3084 RV: ATTTCTCGAGCTGCCTCAGCCTCCTGAGTA;

MDA-MB-231 cells were transfected using Lipofectamine 2000 (Invitrogen) with pGL3-basic vector, pGL3-SPAG5 657–884, and pGL3-SPAG5 2909–3084, in 24-well plates. Firefly and Renilla luciferase activities were measured 72 h post transfection using the Dual-Luciferase Reporter Assay System (Promega) in the GloMax 96 Microplate Luminometer (Promega). Renilla luciferase was used to normalize the Firefly luciferase.

All reactions were performed in triplicate.

### cDNA synthesis and qRT-PCR

One microgram of total RNA was reverse-transcribed at 37 °C for 60 min in the presence of random hexamers and Moloney murine leukemia virus reverse transcriptase (Invitrogen). Specific oligonucleotide primers for GAPDH FW: GAGTCAACGGATTTGGTCGT, RV: GACAAGCTTCCCGTTCTCAG, SPAG5 FW: ACTGAGAGTGATGTTCCTGGA, RV: CTAACTCCTTGTCAGAGCGC were used for PCR analyses. Gene expression levels were measured by quantitative real-time PCR using the SYBR Green assay (Applied Biosystems) on a StepOne instrument (Applied Biosystems). Small amounts of RNA (10 ng) were reverse-transcribed using the TaqMan microRNA Reverse Transcription Kit (Applied Biosystems) in a final volume of 10 µl using an ABI Prism 7000 Sequence Detection System (Applied Biosystems). The PCR reactions were initiated with a 10-min incubation at 95 °C followed by 40 cycles of 95 °C for 15 s and 60 °C for 60 s. qPCR quantification of miRNA expression was performed using TaqMan MicroRNA^®^ Assays (Applied Biosystems) according to the manufacturer’s protocol. RNU6B was used as an endogenous control to normalize miRNA expression.

All reactions were performed in triplicate.

### Lysate preparation and immunoblotting analyses

Cells were lysed in buffer with 50 mM Tris-HCl, pH 8, 150 mM NaCl, 5 mM EDTA, and 1% NP-40 (Igepal AC-630). Extracts were centrifuged at 14,000 rpm for 10 min to remove cell debris. Protein concentrations were determined by colorimetric assay (Bio-Rad). Western blotting was performed using the following primary antibodies: rabbit polyclonal anti-SPAG5 A301-512A Bethyl Laboratories, Inc. (Montgomery, TX), rabbit polyclonal anti-SPAG5 antibody 60940 (Cell Signaling), rabbit monoclonal anti-TAZ HPA007415 (Sigma-Aldrich), rabbit polyclonal anti-YAP PA1-46189 (Invitrogen), mouse monoclonal anti-TEF-1 610922 (BD Biosciences), rabbit monoclonal anti-cyclin A [E23.1] (ab38) (Abcam), rabbit monoclonal anti-cyclin B ab215436 (Abcam), mouse monoclonal anti-cyclin D1 sc-8396 (Santa Cruz), mouse monoclonal anti-cyclin D3 sc-56308 (Santa Cruz), rabbit monoclonal anti-N-cadherin sc-8424 (Santa Cruz), mouse monoclonal anti-Vimentin sc-6260 (Santa Cruz), mouse monoclonal anti-tubulin ab56676 (Abcam), mouse monoclonal anti-Gapdh sc-47724 (Santa Cruz), and rabbit monoclonal anti-p21 2947 (Cell Signalling). Secondary antibodies used were goat anti-mouse, goat anti-rabbit conjugated to horseradish peroxidase (Amersham Biosciences, Piscataway, NJ, USA). Immunostained bands were detected by the chemiluminescent method (Uvitec Alliance, Cambridge).

### Cell treatments

In total, 10^5^ breast cancer cell lines were plated in 60-mm dishes. After 24 h, cells were treated with Dasatinib S5254 (Selleck Chemicals) at a final concentration of 0.05–0.1 µM, Verteporfin S1786 (Selleck Chemicals) at a final concentration of 2 µM, or 50 µg/ml of Agave compound [32] (Aboca Society) for 72 h. Mitomycin M4287 (Merck KGaA, Darmstadt, Germany) at a final concentration of 10 µg/ml was added to the culture medium for incubation at 37 °C for 2 h.

### Colony-formation assay

Breast cancer cell lines were grown at 70% confluence and transfected with Pre-miRNA Precursor-Negative Control (Ambion) and Pre-miRNA10b-3p (Ambion) or siRNAs using *Lipofectamine RNAi* MAX (Invitrogen) according to the manufacturer’s instructions. After 48 h, detached cells were seeded at 500 cells for six-well dishes (Corning-Costar, Tewksbury, MA, USA). Fresh media (25%) was added every 3 days. After 7–10 days, colonies were stained with crystal violet and colonies counted.

### Cell proliferation assay

Cell proliferation was determined by viable cell counting. We seeded 1 × 10^4^ cells in 60-mm plates in duplicate and grew for 96 h. Cell counting was performed after 24, 48, and 72 h of mixing an aliquot of cells 1:1 with Trypan Blue dye (Invitrogen).

### Transwell migration assays

Transfected cells were detached and counted. A migration assay was performed using a 24-well plate. We seeded 5 × 10^4^ cells, in a volume of 500 µl of DMEM without FBS, in the upper chamber with an 8-mm pore-size filter (BD Falcon, Franklin Lakes, NJ, USA), while the bottom chamber of the transwell was filled with 700 µl of DMEM with 10% FBS. Cells were allowed to migrate for 24 h in a humidified incubator at 37 °C and 5% CO_2_. Migrated cells, which had attached to the outside of the filter, were visualized by staining with DAPI (Sigma-Aldrich) and counted under a Zeiss LSM 510 laser-scanning fluorescence confocal microscope.

### Cell viability assay

In all, 8 × 10^2^ cells were seeded into 96-well plates. Cell viability was assessed using ATPlite assay (Perkin Elmer, Massachusetts, USA) according to the manufacturer’s instructions.

### Promoter analysis

FASTA sequences of human SPAG5 (NM 06461) promoter (5000 bp upstream of the TSS) were downloaded from UCSC Genome Browser online database and pasted into LASAGNA-Search 2.0 to identify predicted transcription factor-binding sites. TRANSFAC matrices were used for the analysis.

### ChIP experiments

We performed 1% formaldehyde cross-linking and ChIP experiments as described [33].

The chromatin solution was immunoprecipitated with mouse monoclonal anti-TEF-1 610922 (BD Biosciences), YAP1 polyclonal antibody PA1-46189 (Invitrogen), and rabbit polyclonal H4Ac 2594 (Cell Signaling). The immunoprecipitations were performed using Pierce ChIP-grade Protein G magnetic beads (Thermo Fisher Scientific). The immunoprecipitated and purified chromatin was subjected to RT-qPCR. The promoter occupancy was analyzed by RT-qPCR using the SYBR Green assay (Applied Biosystems). Normalization was performed to the amount of input chromatin.

## Results

### MiR-10b-3p downregulation is anticorrelated to SPAG5 expression in breast cancer tissues

**Fig. 1:**
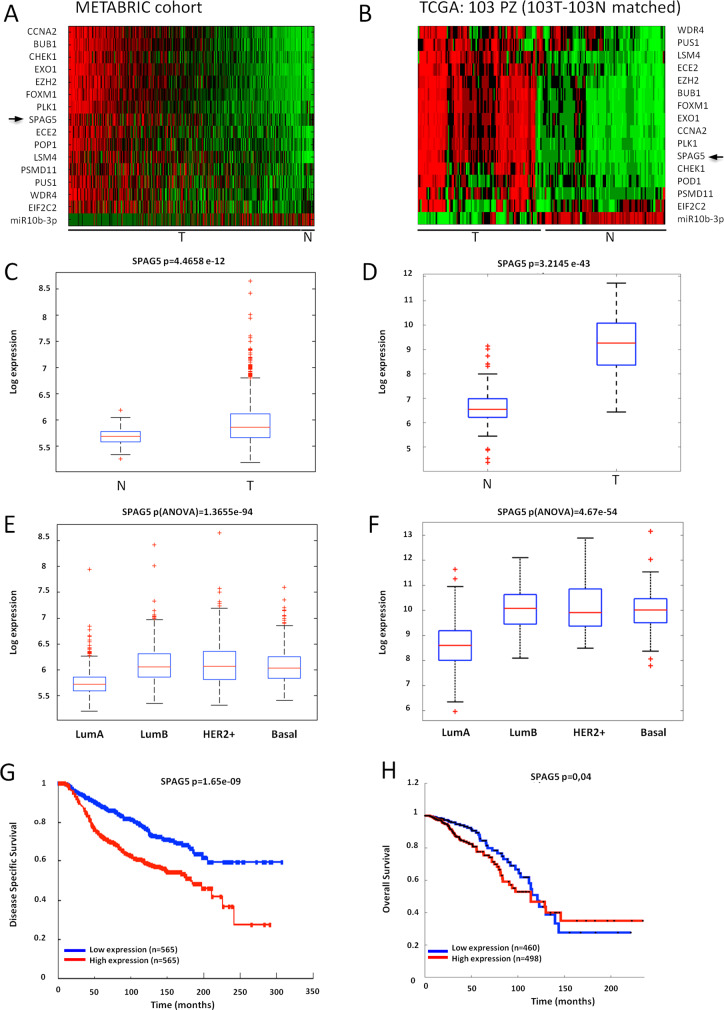
SPAG5 is overexpressed in breast cancer tissues and anticorrelated with miR-10b-3p. **A**, **B** Heatmaps SPAG5 and miR-10b-3p expression in METABRIC cohort (**A**) and in TCGA dataset (**B**). **C**, **D** Boxplot SPAG5 expression in tumoral versus normal breast tissues in METABRIC cohort (**C**) and in TCGA dataset (**D**). **E**, **F** Boxplot SPAG5 expression in Lum-A, Lum-B, Her2, and basal-like breast cancer histotypes in METABRIC cohort (**E**) and in the TCGA dataset (**F**). **G, H** Kaplan–Meier plots show survival analysis of breast cancer patients with low and high expression of SPAG5 in METABRIC cohort (**G**) and in the TCGA dataset (**H**).

We previously demonstrated that forced downregulation of miR-10b-3p in breast cancer perturbed BUB1, PLK1, and CCNA2 expression, hence accelerated tumor progression [34]. Herein, we examined expression levels of miR-10b-3p in two different cohorts of breast cancer patients, 1286 primary breast tumors from the METABRIC dataset, and 103 tumoral and nontumoral matched tissues from the TCGA dataset (Supplementary Fig. 1A, B). In addition, using the CoSMic algorithm [27] and the miRanda tool (http://www.ebi.ac.uk/enright-srv/microcosm/htdocs/targets/v5/), we identified 15 context-specific target genes in the METABRIC dataset as significantly anticorrelated with miR-10b-3p expression (Fig. 1A) [25, 26]. MiR-10b-3p was identified as having a significant anticorrelation (*P* value = 9.86 × 10^−10^/q value = 1.95 × 10^−4^), with 15 target genes (listed in the order of significance) FOXM1, BUB1, EZH2, PLK1, CCNA2, EXO1, ECE2, SPAG5, POP1, PSMD11, PUS1, CHEK1, WDR4, EIF2C2, and LSM4 (Fig. 1B). Among them, we focused on sperm-associated antigen 5 (SPAG5), a recently described promoter of breast tumor growth [19] and a poor prognosis marker of breast cancer [18]. Correspondingly, SPAG5 is strongly expressed (*t* test *P* value = 4.4658e^−12^ and *t* test *P* value = 3.2145 e^−43^) in breast cancer specimens compared to nontumoural tissues, in both METABRIC and in the TCGA dataset (Fig. 1C, D). Interestingly, SPAG5’s expression is higher (ANOVA test *P* = 1.3655e^−94^ and ANOVA test *P* = 1.3655e^−94^) in the Luminal B, HER2, and basal-like breast cancer subtypes, as compared to the Luminal A subtype (Fig. 1E, F). Moreover, SPAG5’s expression significantly associated with shorter patient overall survival in both datasets (Fig. 1G, H). In addition, SPAG5 expression and miR-10b-3p levels were significantly anticorrelated (Fig. 1A, B and Supplementary Fig. 1C, D). In conclusion, our observations raised the possibility that miR-10b-3p directly regulates transcripts encoding SPAG5, a gene associated with the aggressive behavior of mammary tumors.

### SPAG5 is a direct target of miR-10b-3p in breast cancer cell lines

**Fig. 2:**
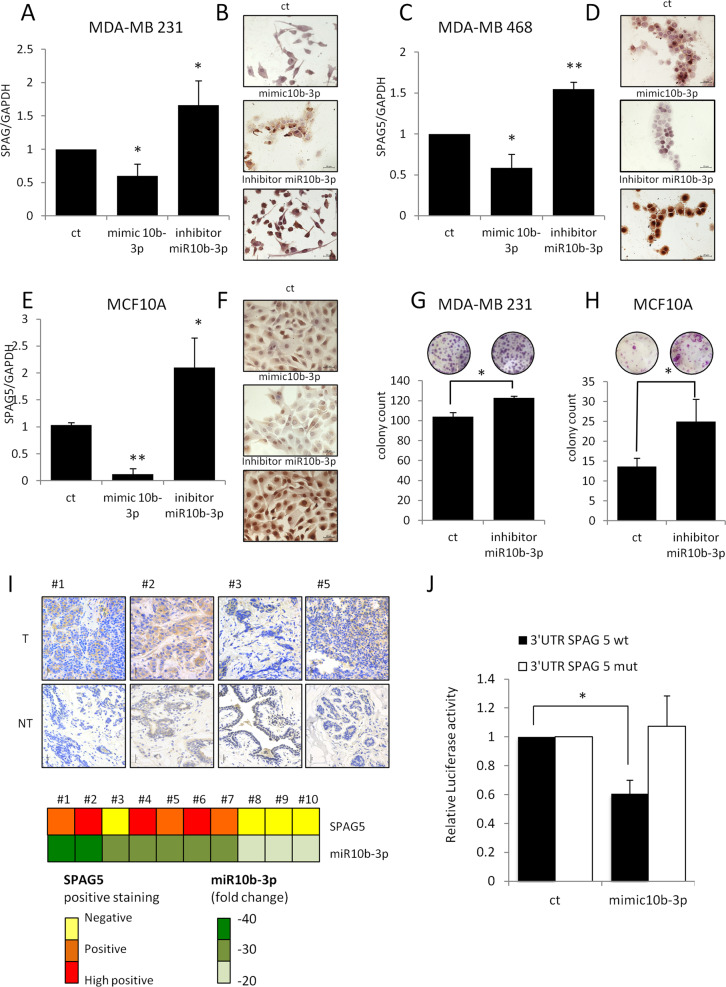
miR-10b-3p affects SPAG5 expression by binding on 3’UTR. **A**, **C**, **E** qPCR expression level of SPAG5 in MDA-MB-231 (**A**), MDA-MB-468 (**C**) breast cancer cell lines, and in MCF10A-untransformed breast cell lines (**E**) assessed by quantitative PCR after 48 h from mimic-10b-3p, miR-10b-3p inhibitor, and negative control transfection. Histograms report the means and *P* value from three independent experiments. **B**, **D**, **F** Immunocytochemistry of positive SPAG5 expression in MDA-MB-231 (**B**), MDA-MB-468 (**D**) breast cancer cell lines, and in MCF10A-untransformed breast cell lines after 48 h from mimic-10b-3p, miR-10b-3p inhibitor, and negative control transfection. **G**, **H** Clonogenic assay of MDA-MB-231 (**G**) and MCF-10A-untransformed breast cell lines (**H**) transfected with miR-10b-3p inhibitor and negative control for 48 h before seeding at clonal density. **I** Immunohistochemistry of SPAG5 expression in a representative matched breast tumor and nontumoral tissues. In the lower panel, the heatmap shows expression levels of miR-10b-3p and SPAG5 for each tumor tissue. SPAG5 level was calculated using *H* score. miR-10b-3p expression was assessed by qRT-PCR. **J** Luciferase assay expression vectors carrying a luciferase reporter followed by the wild-type or mutated 3′-UTR regions of SPAG5 were transfected in HEK-293T cells in the presence of mimic-10b-3p and negative control. Normalized luciferase-activity values and *P* value from three independent experiments are shown (*P value <0.05; **P value <0.001).

In order to assess the direct association of miR-10b-3p with SPAG5 expression, we first located the miR-binding site on the 3′UTR of SPAG5 using the TargetScan Prediction tool (Supplementary Fig. 2A). Ectopic expression of miR-10b-3p reduced mRNA (Fig. 2A–C and Supplementary Fig. 2E–G) and protein levels of SPAG5 in four different breast cancer cell lines: MDA-MB-231, MDA-MB-468, MCF-7, and SUM-159PT (Fig. 2B–D and Supplementary Fig. 2B, C, F–H), respectively, as well in MCF-10A, an untransformed breast cell line (Fig. 2E, F and Supplementary Fig. 2D). Conversely, loss of function of miR-10b-3p by using a specific miRNA inhibitor released SPAG5 transcript (Fig. 2A–C) and protein expression in both MDA-MB-231 and MDA-MB-468 breast cancer cell lines (Fig. 2B–D and Supplementary Fig. 2B, C) and in MCF-10A cells (Fig. 2E, F and Supplementary Fig. 2D). Functionally, the clonogenic ability of MDA-MB-231 breast cancer cell line was slightly increased by inhibition of miR-10b-3p expressions, while a more evident effect was seen in MCF-10A cells (Fig. 2G, H).

We also found that in matched breast tumor and nontumor tissues, lower levels of miR-10b-3p were associated with higher SPAG5 protein expression (Fig. 2I upper and lower panels). Next, to determine whether the predicted binding site within 3′UTR of SPAG5 is directly targeted by miR- 10b-3p, we performed dual Luciferase reporter assay by transfecting cells with plasmids harboring either wild-type SPAG5 or SPAG5 with a deletion at positions 174–176 in the 3′UTR. MiR-10b-3p ectopic expression suppressed luciferase reporter activity of wild-type SPAG5, while it was not decreased in cells transfected with SPAG5 harboring the indicated 3′UTR deletion, see Fig. 2J. Taken together, these results validate that SPAG5 serves as a direct target of miR-10b-3p in breast cancer cells.

### Depletion of SPAG5 expression impairs proliferation and migration of breast cancer cell lines

**Fig. 3:**
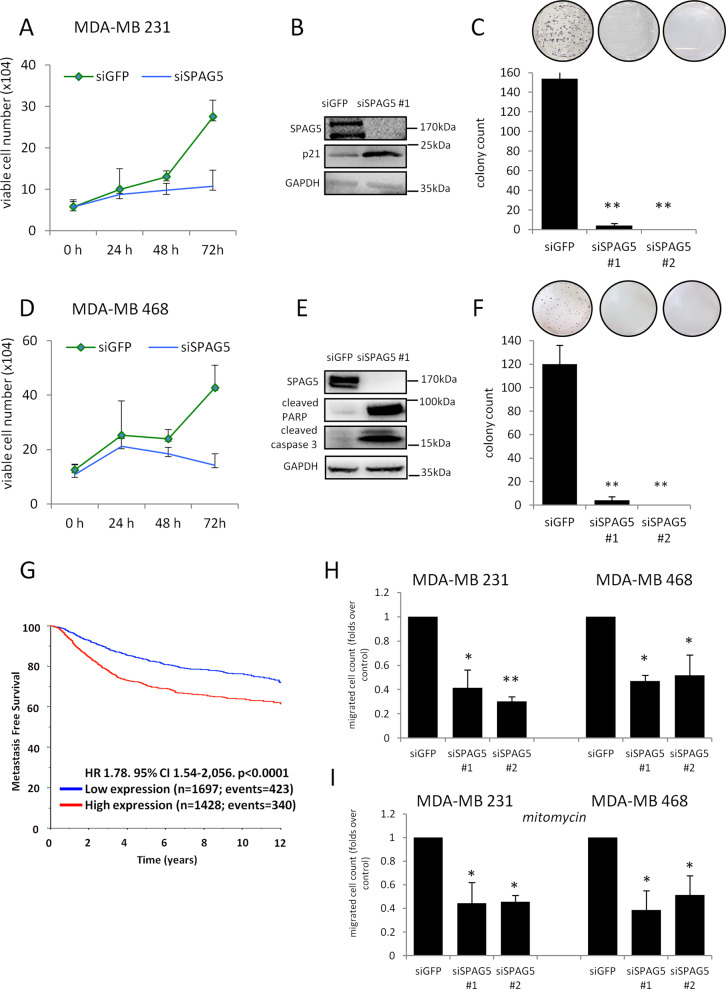
SPAG5 depletion affects proliferation, clonogenicity, and migration of breast cancer cells. **A**–**D** Cellular growth curves of MDA-MB-231 (**A**) and MDA-MB-468 (**D**) breast cancer cell lines determined by counting dye method after 24–48–72 h from siGFP and siSPAG5 tranfection. **B**–**E** Western blot with SPAG5 and p21 antibody of whole-cell lysates of MDA-MB-231 (**B**) breast cancer cell lines and cleaved-PARP and caspase 3 antibodies of whole-cell lysates of MDA-MB-468 (**E**) breast cancer cell lines harvested after 48 h from siGFP or siSPAG transfection. **C**–**F** Clonogenic assay of MDA-MB-231 (**C**) and MDA-MB-468 (**F**) breast cancer cell lines transfected with siGFP and siSPAG5 for 48 h before seeding at clonal density. **G** Kaplan–Meier plots show metastasis-free survival analysis of breast cancer patients with low and high expression of SPAG5. **H**–**I** Migration-assay histograms show the migrated cell count from MDA-MB-231 and MDA-MB-468 breast cancer cell lines determined by transwell assay after 72 h from siGFP or siSPAG5 transfection (**H**) and treated with 10ug/ml of mitomycin (**I**) (*P value <0.05; **P value <0.001).

To assess the activity of SPAG5, we performed loss-of-function assays using siRNA-mediated targeting of SPAG5 in four different breast cancer cell lines: MDA-MB-231, MDA-MB-468, MCF-7, and SUM-159PT. As shown in Fig. 3A–C and D–F and in Supplementary Fig. 3A–D, breast cancer cell proliferation and clonogenicity were significantly reduced in siSPAG5-transfected cells compared with siGFP (control)-transfected cells. MDA-MB-468 and MDA-MB-231 breast cancer cells depleted for SPAG5 protein expression accumulated preferentially in the G2 phase of the cell cycle (Supplementary Fig. 3G). This paired with the increased levels of the CDK inhibitor p21 (Fig. 3B and E and Supplementary Fig. 3C–F). Because high SPAG5 expression associates with shorter metastasis-free survival in breast cancer patients (Fig. 3G), we determined the effect of SPAG5 on breast cancer cell migration. To this end, we carried out transwell assays that employed MDA-MB-231 and MDA-MB-468 cell lines, which were pretreated with siRNAs in a way that depleted SPAG5 expression. As shown in Fig. 3H, I, siSPAG5-transfected cells markedly reduced migration ability. This effect appears to be uncoupled from cell proliferation rate as evidenced by mitomycin treatment (Fig. 3H, I). In addition, in both MDA-MB-231 cells and in MDA-MB-468 cells, the expression of vimentin and N cadherin declined upon SPAG5 depletion (Supplementary Fig. 3H, I). Altogether, these findings suggest that SPAG5 downregulation, using RNA interference, can retard cell proliferation, migration, and EMT.

### SPAG5 overexpression confers oncogenic potential to MCF-10A cells

**Fig. 4:**
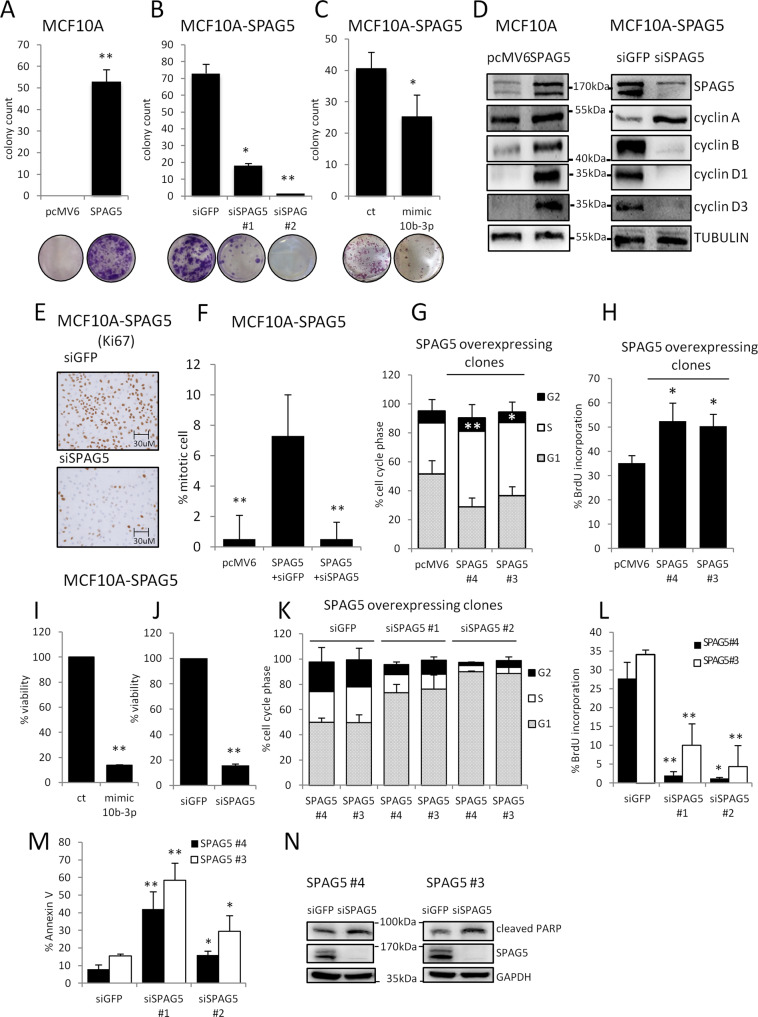
Ectopic expression of SPAG5 confers tumorigenic potential to untransformed MCF-10A cells. **A**–**C** Clonogenic assay. Representative micrographs of colonies formed by MCF-10A cells stably overexpressing either pcMV6 or SPAG5-expression vector (**A**) transfected with siGFP and siSPAG5 (**B**), mimic-10b-3p, and negative control (**C**) for 48 h before seeding at clonal density. Column graphs show colony count and *P* value from three independent experiments. **D** Western blot with SPAG5, cyclin A, cyclin B, cyclin D1, and cyclin D3 of whole-cell lysate of MCF-10A cells stably overexpressing either pcMV6 or SPAG5 (left panel) and MCF-10A cells stably overexpressing SPAG5 transfected with siGFP or siSPAG5 (right panel). **E** Immunocytochemistry of positive Ki-67 expression in MCF-10A cells stably overexpressing SPAG5 transfected with siGFP or siSPAG5. **F** Percentage of mitotic cell**:** the graph shows the percentage of mitotic cells in MCF-10A cells stably overexpressing either pcMV6 and SPAG5 transfected with siGFP and siSPAG5. **G** Cell cycle analysis of MCF-10A cells stably overexpressing either pcMV6 or SPAG5-expressing vector assessed by flow-cytometry assay. **H** BrdU staining of MCF-10A cells stably overexpressing either pcMV6 or SPAG5. **I**–**J** Viability assay of MCF-10A cells stably overexpressing SPAG5 transfected with siGFP or siSPAG5 (**I**) and mimic-10b-3p and negative control (**J**). **K** Cell cycle analysis of MCF-10A cells stably overexpressing SPAG5 transfected with siGFP or siSPAG5 assessed by flow-cytometry assay. **L** BrdU staining of MCF-10A cells stably overexpressing SPAG5 transfected with siGFP or siSPAG5. **M** Annexin V staining of MCF-10A cells stably overexpressing SPAG5 transfected with siGFP or siSPAG5. **N** Western blot cleaved-PARP level in MCF-10A cells stably overexpressing SPAG5 transfected with siGFP or siSPAG5. Data are presented as the mean of three independent replicates (*P value <0.05; **P value <0.001).

To further investigate the oncogenic role of SPAG5 in breast cancer, we set up its stable overexpression in MCF-10A cells. We isolated stably transfected cell clones overexpressing SPAG5 (Supplementary Fig. 4A). The efficiency of colony formation was significantly (*t-*test *P* = 7.92 e^−05^) increased for MCF-10A cell clones overexpressing SPAG5 compared to control cells (Fig. 4A and Supplementary Fig. 4B). In addition, we investigated the impact of either SPAG5 depletion (Fig. 4B and Supplementary Fig. 4C) or ectopic expression of miR-10b-3p on MCF-10A/SPAG5 cell clones (Fig. 4C and Supplementary Fig. 4D). Clear reductions in the number of colonies were revealed under both experimental conditions, as compared to control cells (Fig. 4B, C). To elucidate the oncogenic activity of SPAG5, we analyzed several cell cycle regulators, namely cyclin A, B, D1, and D3 in MCF-10A cells overexpressing SPAG5. Consistently, elevated levels of all four cyclins were observed in MCF-10A/SPAG5 clones compared to control cells (Fig. 4D). Furthermore, immunostaining of the cellular proliferation marker Ki-67 [35, 36] was strongly increased in clones overexpressing SPAG5 (Fig. 4E).

SPAG5 was first described as a dynamic regulator of the mitotic spindle, chromosome segregation, and mitotic progression [7, 8, 10, 11, 37]. It has been reported that SPAG5 interference exerts no effect on the distribution and morphology of mitotic microtubules rather the generation of multipolar and highly disordered mitotic spindles, which drove growth arrest [8]. Accordingly, γ-tubulin staining highlighted large amounts of mitotic cells in MCF-10A cells overexpressing SPAG5 and no perturbation of the spindle microtubules; instead, SPAG5 depletion affected chromosome alignment and impaired mitosis (Fig. 4F and Supplementary Fig. 4J). Likewise, depletion of SPAG5 in the SUM-159PT triple-negative breast cancer cell line markedly reduced cell cycle-associated proteins, such as cyclin A, cyclin B (Supplementary Fig. 4E), and the cellular proliferation marker Ki-67 (Supplementary Fig. 4F), as well as counteracts mitotic spindle formation (Supplementary Fig. 4G).

Cell cycle analysis of different MCF-10A/SPAG5-overexpressing clones and control cells evidenced active DNA synthesis and increased S-phase fraction of proliferating cells (Fig. 4G, H). Viability assay further validated the oncogenic potential of SPAG5, which was counteracted either by miR-10b-3p overexpression or by SPAG5 depletion (Fig. 4I, J) in line with the results from SUM-159PT triple-negative breast cancer cell line (Supplementary Fig. 4H, I). Notably, depletion of SPAG5 halted the proliferation and cell cycle progression reduced de novo DNA synthesis, and increased the apoptotic rate of MCF-10A/SPAG5 clones (Fig. 4K–N).

Taken together, these results highlight an important role for SPAG5 as a regulator of cell cycle progression and proliferation.

### The transcriptional axis YAP/TAZ/TEAD controls SPAG5 expression in breast cancer cells

**Fig. 5:**
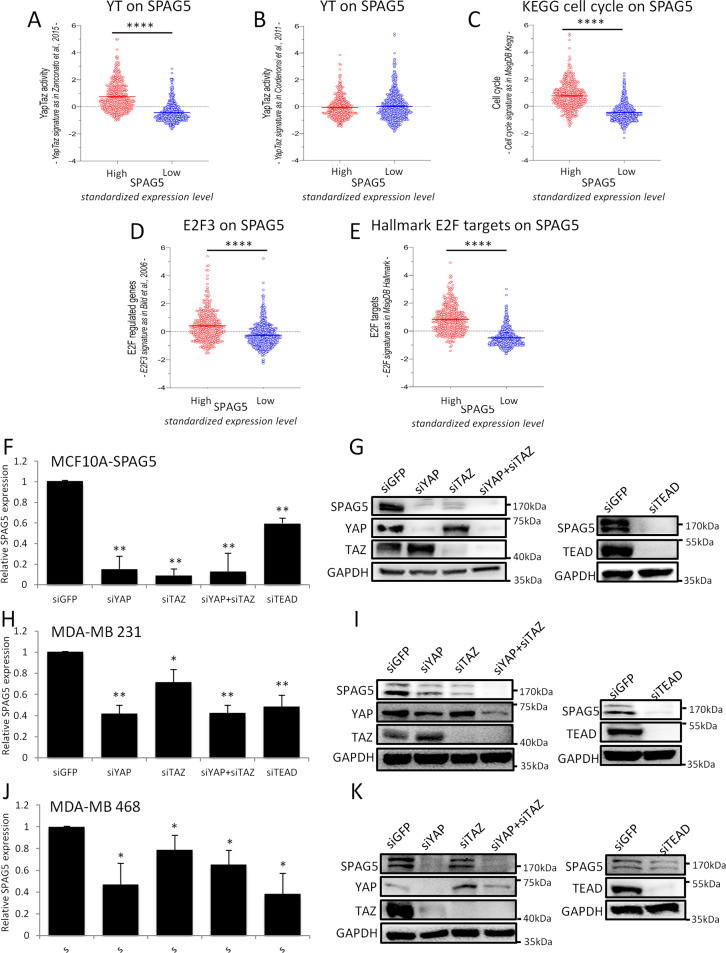
YAP/TAZ signature score in high and low SPAG5 breast cancer samples. **A** SPAG5 expression in YAP/TAZ signature from [42]. **B** SPAG5 expression and YAP/TAZ signature from ref. [30]. **C** SPAG5 expression in cell cycle signature from MsigDB KEGG. **D** SPAG5 expression in E2F3 signature from ref. [43]. **E** E2F signature in MsigDB Hallmark. Unpaired *t*-test *P* value<0.0001. Selective depletion of YAP. TAZ, TEAD reduces SPAG5 expression. **F**, **H**, **J** qPCR expression level of SPAG5 in MCF-10A cells stably overexpressing SPAG5 (**F**) in MDA-MB-231 (**H**) and in MDA-MB-468 (**J**) assessed by quantitative PCR after 48 h from siGFP, siYAP, siTAZ, and siTEAD transfection. **G**, **I**, **K** Western blot shows SPAG5 protein level in MCF-10A cells stably overexpressing SPAG5 (**G**), in MDA-MB-231 (**I**) and in MDA-MB-468 (**K**) after 48 h from siGFP, siYAP, siTAZ, and siTEAD transfection (*P value <0.05; **P value <0.001).

The involvement of the Hippo pathway in cancer occurrence, especially deregulation in breast cancer [23, 38–40], is well established. Importantly, elevated expression of YAP/TAZ signature correlates with high histological grade, enrichment of stem cells, metastasis, and resistance to Taxol [22, 41]. Intriguingly, high levels of SPAG5 transcript expression in breast cancer patients exhibit a statistically significant association with elevated YAP/TAZ signature mainly enriched for target genes involved in cell cycle regulation as from Zanconato et al. [42] (Fig. 5A). No statistically significant association was found with YAP/TAZ signature from Cordenonsi et al. whose gene target composition is broader than that of Zanconato et al., and not specifically enriched for cell cycle genes [30] (Fig. 5B). Furthermore, SPAG5 expression levels associate with known cell cycle-enriched gene signatures, such as from MsigDB KEGG pathway, E2F2, and E2F3 target gene signatures (Fig. 5C–E) [43]. Collectively, these data indicated SPAG5 as a YAP/TAZ cell cycle-related target gene.

Second, we depleted (using siRNAs) the expression of three main effectors of the pathway, YAP, TAZ, and TEAD, in MCF-10A cells stably overexpressing SPAG5, and in three different triple-negative breast cancer cell lines, MDA-MB-231, MDA-MB-468, and SUM-159PT. Reassuringly, SPAG5 transcripts were significantly reduced 48 h after siRNA transfection (Fig. 5F, H–J), while SPAG5 protein declined 24 h later (Fig. 5G, I, K and Supplementary Fig. 5A). No significant perturbation of miR-10b-3p levels was attested (Supplementary Fig. 5B, C), suggesting direct transcriptional control of SPAG5 promoter by TEAD, YAP, and TAZ.

### SPAG5 is a direct transcriptional target of TEAD

**Fig. 6:**
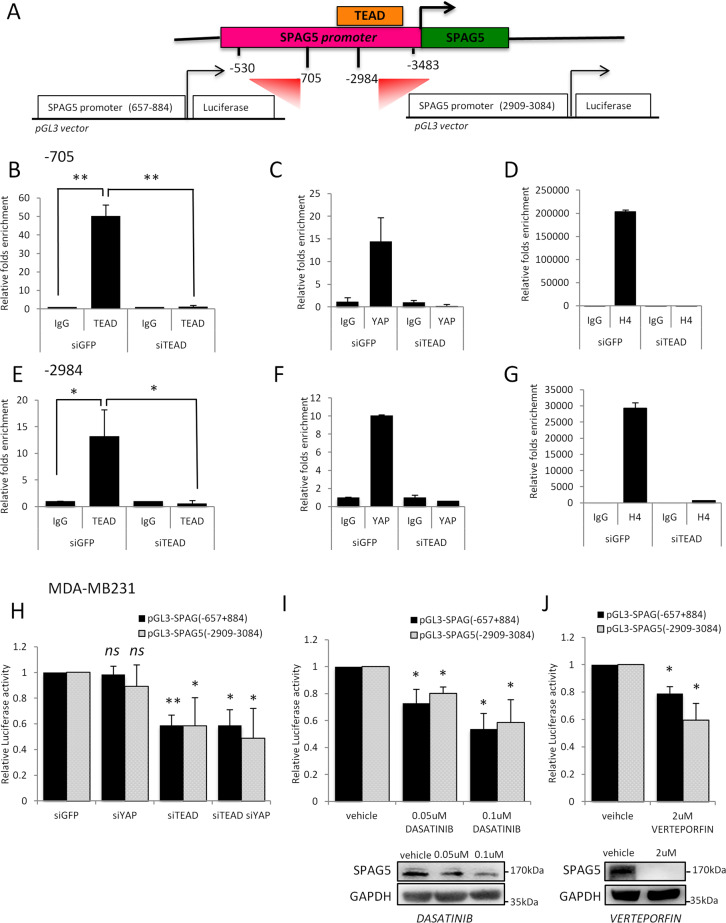
TEAD and coactivator YAP directly bind the SPAG5 promoter. **A** Schematic representation of the SPAG5 promoter with the putative TEAD-binding sites (LASAGNA Search 2.0). The promoter regions 657–884 and 2909–3084 were amplified and cloned into pGL3 vector. **B**, **C**, **E**, **F** Chip analysis of the TEAD and YAP binding on SPAG5 promoter in MDA-MB-231 cell line after TEAD interference detected by qRT-PCR analysis. **D**–**G** Transcriptional active chromatin on SPAG5 promoter evidenced by anti-H4-Acetylate antibody. Data are shown as the mean of three independent replicates with the a relative *P* value. **H**–**J** Luciferase assay pGL3 vector carrying SPAG5 657–884 and SPAG5 2909–3084 promoter regions was transfected in MDA-MB-231 cell line after YAP and/or TEAD interference. **H** MDA-MB-231 cell lines were treated with 0.05–0.1 µM of Dasatinib (**I**) or with 2 µM of Verteporfin (**J**) and transfected with pGL3 vector carrying SPAG5 657–884 and SPAG5 2909–3084 promoter regions. Normalized luciferase-activity values and *P* value from three independent experiments are shown. In the lower panel, western blot shows SPAG5 expression after 0.05–0.1 µM of Dasatinib (**I**) or with 2 µM of Verteporfin (**J**) treatments. (*ns* nonsignificant; *P value <0.05; **P value <0.001).

Currently, the transcriptional regulation of SPAG5 is poorly understood. Using the LASAGNA algorithm (Length-Aware Site Alignment Guided by Nucleotide Association), we searched for TEAD1-binding sites on the SPAG5 promoter (Fig. 6A). To validate the in silico analysis for TEAD- binding sites on SPAG5 promoter, we performed ChIP assays in MDA-MB-231 following depletion of TEAD expression (Supplementary Fig. 6A, B). CTGF, a canonical transcriptional target of the Hippo pathway, was used as a positive control (Supplementary Fig. 6C, D). These assays found a significant enrichment of TEAD and YAP recruitment on selected regions of SPAG5 promoter (Fig. 6B, C, E, F, and Supplementary Fig. 6E, F, H, I). This paired with increased Histone 4 acetylation, a marker of transcriptionally active chromatin (Fig. 6D, G and Supplementary Fig. 6G–J). Accordingly, TEAD depletion (Supplementary Fig. 6A, B) markedly reduced both TEAD and YAP recruitment onto the SPAG5 promoter and the histone acetylation of the selected regions (Fig. 6B–G and Supplementary Fig. 6E–J).

To further establish the transcriptional control of TEAD on the SPAG5 promoter, we performed transactivation assays. Promoter regions encompassing TEAD-binding sites were cloned in front of a luciferase gene reported (pGL3-SPAG5 657–884 and pGL3-SPAG5 2909–3084). This experimental approach revealed that the depletion of TEAD expression can reduce SPAG5 promoter activity cloned regions (Fig. 6H), and this was further strengthened by YAP codepletion. Collectively, these findings show that SPAG5 is a direct transcriptional target of TEAD.

### Pharmacological targeting of YAP/TAZ impairs SPAG5 oncogenic activity in triple-negative breast cancer cells

The advanced anticancer therapies inhibiting YAP stabilization, nuclear localization, and interaction with TEAD bear the therapeutic potential for tumors with aberrant Hippo activation [44]. To directly examine this prediction, we tested whether the clinically approved YAP and TAZ inhibitors, Dasatinib and Verteporfin, can affect the transcriptional effect of YAP-TAZ and TEAD on SPAG5 promoter fragments. Indeed, we found that increasing doses of Dasatinib and Verteporfin reduced pGL3-SPAG5 657–884 and pGL3-SPAG5 2909–3084 promoter fragments measured as luciferase activity (Fig. 6I, J). Of note, treatment of MDA-MB-231 breast cancer cells and MCF-10A cells stably overexpressing SPAG5 with Dasatinib and Verteporfin reduced SPAG5 protein level in a dose-dependent manner (Fig. 6I, J lower panel and Supplementary Fig. 6L, N, O) and affected their respective colony-forming ability (Supplementary Fig. 6K–M).

Yet another potential drug, Agave, is a natural compound with antimicrobial, pro-apoptotic, immunomodulatory, antiproliferative, and anti-migratory properties. We previously reported that Agave exerts its anticancer activity in part by impairing YAP’s and TAZ’s pro-tumorigenic activities, both transcriptionally and post transcriptionally [32]. Accordingly, we tested whether treatment with Agave can affect SPAG5 expression in MDA-MB-231 and in MDA-MB-468 breast cancer cell lines. Indeed, we found that Agave markedly reduced SPAG5 mRNA expression (Supplementary Fig. 6P–R) and protein expression (Supplementary Fig. 6Q–S) in both breast cancer cell lines. Being a natural compound with ascertained multitargeting activity, Agave might also impact on SPAG5 expression triggering additional signaling pathways than YAP/TAZ/TEAD axis modulation [32].

Collectively, these findings lend further support to the role played by YAP and TAZ in the control of SPAG5 expression.

### The combination of YAP signature and SPAG5 expression predicts breast cancer mortality

**Fig. 7:**
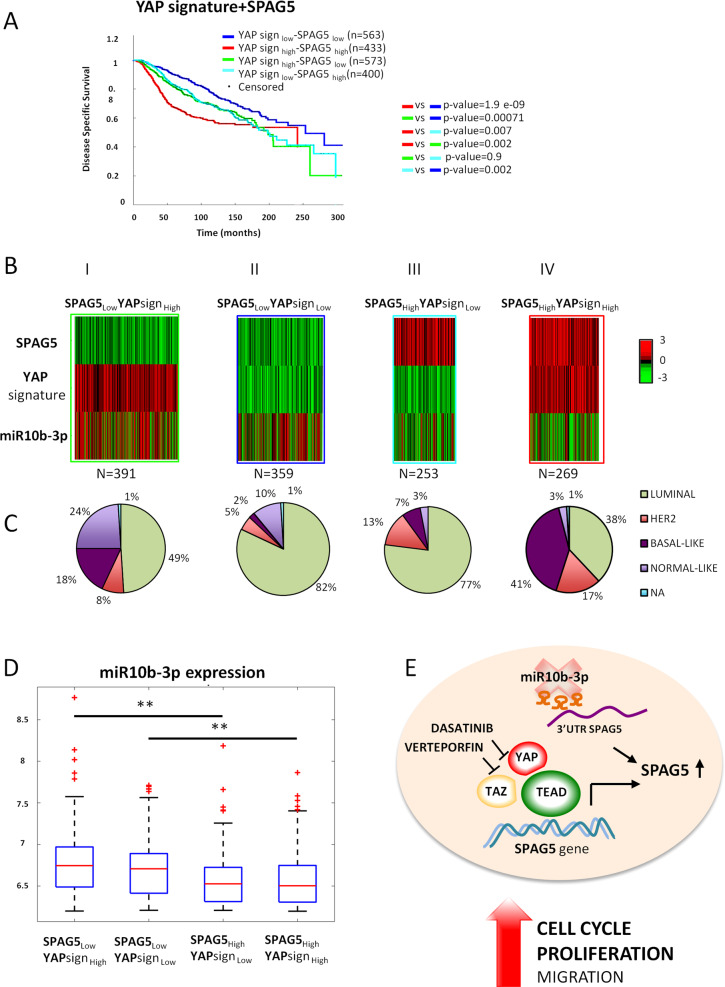
YAP signature and SPAG5 are prognostic independent factors for disease-specific survival of breast cancer METABRIC patient dataset. **A** Kaplan–Meier curve for disease-specific survival on breast cancer patients stratified for different combined levels of YAP signature and SPAG5 expression. Cox regression multivariate analysis adjusted for T, N, stage, istotype, menopause, and age. **B** Heatmaps of standardized expression levels of SPAG5, YAP signature, and miR-10b-3p on breast cancer Metabric dataset. **C** Pie-chart breast cancer histotypes in the different groups of breast cancer patients stratified for combined levels of YAP signature SPAG5 and miR-10b-3p expression. **D** Boxplots of miR-10b-3p expression in Metabric breast cancer patient dataset stratified for different combined levels of YAP signature and SPAG5 expression. **E** Proposed model depicting the transcriptional and post-transcriptional modulation of SPAG5 expression in breast cancer (*P value <0.05; **P value <0.001).

In order to support the independent role of YAP-TAZ-TEAD axis and SPAG5 in breast cancer development, we conducted an analysis of data from the METABRIC dataset. METABRIC is a large- cohort prospective study of very well molecularly characterized breast cancer cases [25]. As a first step, we performed univariate analysis to test whether YAP signature and SPAG5 expression were actually prognostic factors for disease-specific survival. To this end, we used the whole Metabric dataset (*N* = 1969 patients) and we confirmed YAP signature and SPAG5 as prognostic factors for breast cancer (Table 1). Subsequently, in the same Metabric cohort (*N* = 1969 patients), we conducted a Cox multivariate analysis to control the results for potential confounding factors, and we observed that both YAP signature and SPAG5 were independent prognostic factors after adjusting for T, N, stage, hystotype, age, and menopausal state (Table 1). In the second step, we subdivided Metabric patients into four groups: YAP_low_ (expression)/SPAG5_low_ (expression), YAP_high_/SPAG5_high_, YAP_high_/SPAG5_low_, and YAP_low_/SPAG5_high_ and found that YAP_high_/SPAG5_high_ group exhibited statistically significant shorter disease-specific survival when compared with the three other selected groups of Metabric patients (Fig. 7A and Table 2). In the third step, we considered in the analysis only those groups of patients characterized by concordant YAP signature and SPAG5 expression (e.g., YAP_high_/SPAG5_high_ and YAP_low_/SPAG5_low_, *N* = 996 patients). This strategy allowed us to find a statistically significant and direct correlation between the two variables for a Pearson’s *R* = 0.61 (*P* = 7.6E–104). We also observed that between the group YAP_high_/SPAG5_high_ and the group YAP_low_/SPAG5_low_, there was the highest difference in disease-specific survival (log-rank test *P* = 1.9×E–09) being the first group characterized by the shortest survival (Fig. 7A). We then analyzed the expression of miR-10b-3p in relation to both YAP signature and SPAG5 expression. In this section of analyses, we selected from the METABRIC breast cancer dataset, the whole group of cases for which data on SPAG5 expression, YAP signature, and miR- 10b-3p expressions were available at the same time from the same breast cancer cases. In order to understand the miR-10-3p expression in relation to both SPAG5 and YAP signature, we then subdivided again the whole group (*N* = 1279) into the four different categories as before, characterized by a different mode of expression of SPAG5 and YAP signature: (1) YAP_high_/SPAG5_high_ and (2) YAP_low_/SPAG5_low_ (representing 1 and 2 the two concordant categories); (3) high YAP_high_/SPAG5_low_ and (4) YAP_low_/SPAG5_high_ (representing 3 and 4 the two discordant categories). In Fig. 7B, C, we described the observed association between the expression of SPAG5, YAP signature, and miRNA10b-3p expression through the different categories and different analytical steps. In Fig. 7B, we observed the heatmap describing miR-10b-3p down-/upregulation in relation to SPAG5, YAP signature expression by the four categories described above. The graph on the extreme right represents the concordant group YAP_high_/SPAG5_high_. Here, the highest level of SPAG5 expression correlates with a strong downregulation of miR-10b-3p corroborating the anticorrelation of miR-10b-3p and SPAG5 expression. Concomitantly, also, YAP signature exhibited its highest level of expression (Fig. 7BIV). The second graph from the right shows again the SPAG5 high expression accompanied with again miR-10-3p downregulation, despite a low expression of YAP signature corroborating the previous evidence of a direct anticorrelation between SPAG5 and miR-10-3p (Fig. 7BIII). In the third graph from the right, we observed the second concordant category (SPAG5_low_/YAP_low_) in which, this time, miR-10b-3p tends to become less downregulated than before, supporting again an initial anticorrelation effect of miR-10b-3p on SPAG5, which appears to be independent of YAP signature expression (Fig. 7BII). Finally, in the fourth graph, the more potent expression of miR-10-3p corresponds to a very low expression of SPAG5 in spite of YAP signature that now is high, indicating a potentially strongest modulation on SPAG5 of miR- 10b-3p in comparison with YAP expression (Fig. 7BI). The last heatmap further confirms the analytical evidence of an independent effect between YAP signature and miR-10b-3p on SPAG5 modulation. Figure 7C also shows the different distribution by histotypes (luminal, Her2, basal-like, and normal-like) of the four concordant/discordant categories. The hystotype characterization supports, as expected, the highest mortality for the more aggressive breast cancer-concordant category (SPAG5_high_-YAP_high_), at high prevalence in basal-like tumors (Fig. 7C). Figure 7D shows quantitatively the expression of miR-10b-3p by the four different groups, with the highest statistically significant difference for the discordant category SPAG5_low_-YAP_high_.

**Table 1:** YAP signature and SPAG5 are independent prognostic factors for breast cancer.

*N* patients = 1969	Cox univariate		Cox multivariate	
	HR [95% CI]	*P* value	HR [95% CI]	*P* value
YAP signature	1.26 [1.16–1.36]	8.9e–09	1.25 [1.15–1.35]	4.026e–08
SPAG5	1.22 [1.13–1.31]	9.47e–08	1.21 [1.12–1.29]	5.37e–07

Cox-univariate and Cox-multivariate analysis for YAP signature and SPAG5 expression on breast cancer Metabric dataset (*N* = 1969 patients).

**Table 2:** Prognosis of highly correlated breast cancer patients with high levels of YAP signature and high levels of SPAG5.

*N* patients = 996	Cox multivariate	
	HR [95% CI]	*P* value
YAPsign_high_ + SPAG5_high_ and YAPsign_low_ + SPAG5_low_	1.88 [1.39–2.53]	3.8e–05

Cox-multivariate analysis of breast cancer Metabric patients with high and low levels of YAP signature and SPAG5 adjusted for T, N, stage, hystotype, age, and menopausal state (*N* = 996 patients).

Collectively, these results indicate the strong interconnection within the network of three prognostic factors, SPAG5, YAP, and miR-10b-3p expression that also pairs with the prevalence of different cell populations in the identified breast cancer subtypes.

## Discussion

It is widely accepted that breast cancer is a heterogeneous disease, which comprises several clinically and morphologically distinct subtypes, differing in genetic and epigenetic alterations that strongly affect diagnosis, treatment, and prognosis. Depicting how tumor cells are able to grow and spread to distant sites will surely contribute to efficaciously treat breast cancer. miRNA engagement in the onset of the malignant state through the tumor progression and metastasis is now extensively ascertained [45, 46]. Here, we identified SPAG5 as a novel miR-10b-3p target gene. Interestingly, high expressions of SPAG5 associate with lower expression of miR-10b-3p in both METABRIC and TCGA breast cancer datasets and correlate with lower disease-specific survival. Aberrant SPAG5 expression has been reported in several cancer types. This appears to be triggered by Ch.17q11.2 locus gain or amplification [18]. SPAG5 knockdown significantly downregulated the activation of Akt signaling. Indeed, downregulation of miR-363-3p allows SPAG5 to exert its oncogenic activity initiating PI3K/AKT pathway via CEP55 interaction in hepatocellular carcinoma [13]. PLK1 is a direct target of miR-10b-3p [34], and it acts as an upstream kinase needed for SPAG5 phosphorylation and kinetochore organization [47]; our results suggest that restoring miR-10b-3p expression might contribute to properly tune mitotic spindle assembly in breast cells. Altogether, these findings indicate that downregulation of miR-10b-3p leads to aberrant cell proliferation by means of unleashing a coordinated network of cell cycle regulators, which impinge on diverse signaling pathways governing cell proliferation. We also document that SPAG5 ectopic expression confers upon untransformed MCF-10A breast cells the ability to form colonies, and, concordantly, SPAG5 depletion markedly reduces proliferation, clonogenicity, and cancer cell migration, as well as affects the main EMT markers, such as vimentin and N cadherin (Fig. 3H, I and Supplementary Fig. 3H, I). Interestingly, high expression of SPAG5 associates with shorter metastasis-free survival in breast cancer patients (Fig. 3G). These findings provide further evidence confirming the oncogenic role of SPAG5 in breast cancer, as well as in other tumor types.

Little is known about SPAG5’s transcriptional regulation. Intriguingly, by integrating specific genes, miRNA expression patterns, and transcriptional profiles, we found that breast cancer patients with high SPAG5 expression exhibit an activated YAP/TAZ signature, implying that aberrant activation of the two key transducers either independently or dependently from their role in the Hippo pathway enhances SPAG5’s oncogenic activity. Accordingly, the depletion of either YAP or TAZ significantly reduced SPAG5 transcript and protein expression. Interestingly, this effect was also highlighted upon depletion of the TEAD transcription factor, suggesting that SPAG5 could serve as a breast cancer target within the oncogenic transcriptional axis YAP/TAZ/TEAD. Specifically, we found that YAP and TEAD binding-to-binding sites were mapped in SPAG5’s promoter paired with increased histone acetylation, a marker of active transcription. Notably, the critical involvement of the YAP/TAZ/TEAD axis as a broad transcriptional regulator of breast cancer tumorigenesis has previously been reported [22, 23, 48–52], but the identification of transcriptional targets with specific activities in breast cancer still needs to be elucidated. Intense research efforts have been focused on the pharmacological targeting of the YAP/TAZ/TEAD transcriptional axis in human cancers. Diverse compounds acting either on YAP/TAZ nuclear localization or reducing protein expression have been identified and hold promise for clinical applications.

Among other experimental approaches, Dasatinib and Verteporfin were shown to strongly impair YAP/TAZ co-transcriptional activities in breast cancer cell lines. Accordingly, we found that the treatment of breast cancer cell lines with either Dasatinib or Verteporfin and with extracts of Agave reduced SPAG5 expression. Dasatinib has recently been challenged in a phase II trial on unselected advanced triple-negative breast cancer patients, exhibiting limited activity as a single agent [53]. Interestingly, there are active clinical trials with Verteporfin for breast cancer and other solid tumors *(NCT02872064, NCT03033225,* and *NCT04590664*). Based on its previously reported role as a determinant of drug response in breast cancer patients, along with its contribution to an improved determination of breast cancer subtypes, SPAG5 might hold the potential to be a promising biomarker, which identifies patients who could benefit from drugs targeting YAP/TAZ activities, either alone or in combination with other treatments.

In summary, our findings identify a novel transcriptional and post-transcriptional network, which leads to aberrant activation of SPAG5 in breast cancer. The timing of this network might be differently regulated within the diverse breast cancer subtypes. The uncovered scenario features both early (post-transcriptional) and late (transcriptional) steps of tumorigenesis, fine resolution of which would allow designing effective therapeutic approaches [45] (Fig. 7E).

## Supplementary information

Legend to Supplementary Figure

Suppl. Fig1

Suppl. Fig 2

Suppl. Fig 3

Suppl. Fig 4

Suppl. Fig 5

Suppl. Fig 6
