# Supplementary material for: Aberrant transcriptional and post-transcriptional regulation of SPAG5, a YAP-TAZ-TEAD downstream effector, fuels breast cancer cell proliferation
Source: Cell Death Differ. 2020 Nov 23;28(5):1493–511. doi: 10.1038/s41418-020-00677-9 (PMC8166963; doi:10.1038/s41418-020-00677-9)
Supplement: Supplementary file 1 — Legend to Supplementary Figure [file 41418_2020_677_MOESM1_ESM.docx]

**Legends to Supplementary Figures**

**Suppl. Fig1: A-B Box-plot** miR 10b-3p expression in tumoral vs non-umoral breast tissues in METABRIC cohort (left panel) and in TCGA dataset (right panel). **C-D Box-plot** miR- 10b-3p expression in Lum-A, Lum-B, Her-2, Basal-like breast cancer hystotype in METABRIC cohort (left panel) and in TGCA dataset (right panel).

**Suppl. Fig2: A.** Schematic representation of the miR 10b-3p binding site on 3’UTR of SPAG5 (www.miRbase.org) **B-D Western blot** with SPAG5 antibody of whole cell lysates of MDA-MB231 (B), MDA-MB468 (C) breast cancer cell lines and in MCF10A (D) un-transformed breast cell line harvested after 48hrs from mimic 10b-3p, miR 10b-3p inhibitor and negative control transfection. **E-G qPCR** expression level of SPAG5 in MCF7 (E) and in SUM-159PT (G) breast cancer cell lines assessed by quantitative PCR after 48hrs from mimic 10b-3p or negative control transfection. Histograms report the means and p-value from three independent experiments. **F-H** (upper panel) **Immunocytochemistry** of SPAG5 expression in MCF7 (F) and SUM-159PT (H) breast cancer cell lines after 48hrs from mimic 10b-3p or negative control transfection and (lower panel) **Western blot** with SPAG5 antibody of whole cell lysates of MCF7 and SUM-159PT breast cancer cell lines harvested after 48hrs from mimic 10b-3p or negative control transfection.

**Suppl. Fig3: A-D Clonogenic assay** of MCF7 (A) and SUM-159PT (D) breast cancer cell lines transfected with siGFP and siSPAG5 for 48hrs before seeding at clonal density. **B-E Cellular growth curves** of MCF7 (B) and SUM-159PT (E) breast cancer cell lines determined by counting dye method after 24-48-72 hrs from siGFP or siSPAG5 transfection. **C-F Western blot** with SPAG5 and p21 antibody of whole cell lysates of MCF7 (C) and SUM-159PT (F) breast cancer cell lines harvested after 48hrs from siGFP or siSPAG5 transfection (*pvalue <0.05 **pvalue <0.001). **G-H Western blot** analysis of the whole cell lysate from MDA-MB231 and MDA-MB468 assessed for SPAG5, E-cadherin and Vimentin expressions harvested after 72hrs from siGFP or siSPAG5 transfection (G) and treated with 10ug/ml of mytomicin (H).

**Suppl. Fig4: A Western-blot** of MCF-10A cells individual clones overexpressing SPAG5 screened for SPAG5 protein expression. Clonogenic assay were performed to assess the acquired oncogenic ability. **B-D Western-blot** SPAG5 protein level of whole cell lysates of MCF-10A cells stably overexpressing pcMV6 or SPAG5 (B) MCF-10A stably overexpressing SPAG5 transfected with siGFP and siSPAG5 (C) and MCF-10A stably overexpressing SPAG5 transfected with mimic 10b-3p and negative control (D). **E Western-blot** with SPAG5, cyclin A, cyclin B, of whole cell lysate from SUM-159PT after 48hrs from siGFP or siSPAG5 transfection. **F Immunocytochemistry** of positive Ki67 expression in SUM-159PT transfected with siGFP and siSPAG5. **G Percentage of mitotic cell:** graph shows the percentage of mitotic cells in SUM-159PT transfected with siGFP and siSPAG5**. H-I Viability assay** of SUM-159PT cells transfected with siGFP or siSPAG5 (H) and mimic 10b-3p and negative control (I). **J Immunofluorescence assay** representative images of mitosis from MCF10A cells stably overexpressing pcMV6 and SPAG5 transfected with siGFP and siSPAG5 for 48hrs, stained with anti-SPAG5, anti γ-tubulin. Nuclei were stained with DAPI. Scale-bar 20um (*pvalue <0.05 **pvalue <0.001).

**Suppl. Fig5: A Western-blot** SPAG5 protein level in SUM-159PT cells after 48hrs from siGFP, siYAP, siTAZ si TEAD transfection **B-C qPCR** Relative expression level of miR 10b-3p in MCF-10A stably overexpressing SPAG5 (B) and in MDA-MB231 breast cancer cells (C) assessed by quantitative PCR after 48hrs from siGFP, si YAP, siTAZ and siTEAD transfection. **D-E** **Clonogenic assay** of MCF-10A cells stably overexpressing SPAG5 (D) and MDA-MB231 breast cancer cells (E) transfected with siGFP, si YAP, siTAZ and siTEAD for 48hrs before seeding at clonal density. Histograms report the means and p-value from three independent experiments (*ns* non significant, *pvalue <0.05 **pvalue <0.001).

**Suppl. Fig 6: A qPCR** expression level of SPAG5 after 40hrs from TEAD interference in MDA-MB231 cell line used for chip analysis. **B Western-blot** protein level of SPAG5 and TEAD in MDA-MB231 cell line used for chip analysis. **C-D qPCR** CHIP analysis of CTGF promoter in MDA-MB231 cells after interference of TEAD in the replicates used for SPAG5 promoter analysis. **E-F and H-I** **qPCR** Chip analysis of the TEAD and YAP binding on SPAG5 promoter in MDA-MB231 cell line after TEAD interference detected by qRT-PCR analysis. **G-J** Transcriptional active chromatin on SPAG5 promoter evidenced by anti-H4-Acetylate antibody. Data are shown as mean of three independent replicates with the relative p-value (*pvalue <0.05 **pvalue <0.001).

**Dasatinib, Verteporfin and Agave treatment affect SPAG5 expression. K-M clonogenic assay** Representative micrographs of colonies formed by MDA-MB231 (K) and MCF-10A cells stably overexpressing SPAG5 (M) treated with 0,05uM-0,1uM of Dasatinib for 72 hrs before seeding at clonal density. Column graphs show colony count and p-value from three independent experiments **L-N Western-blot** SPAG5, YAP and TAZ protein levels after 72 hrs with 0,05uM-0,1uM of Dasatinib treatment in MDA-MB231 (L) and MCF-10A cells stably overexpressing SPAG5 cell lines (N). **O Western blot** SPAG5, YAP and TAZ protein levels after 72 hrs of treatment with 2uM of Verteporfin in MDA-MB231. **P-R qPCR** SPAG5 expression after 72 hrs of 50 ug of Agave treatment in MDA-MB231 (P) and MDA-MB468 (R) cell lines. **Q-S Western-blot** SPAG5, YAP, TAZ and TEAD protein levels after 72 hrs of 50 ug of Agave treatment in MDA-MB231 (Q) and MDA-MB468 (S) cell line. (*pvalue <0.05 **pvalue <0.001).
