# Supplementary figures and images for: Aberrant transcriptional and post-transcriptional regulation of SPAG5, a YAP-TAZ-TEAD downstream effector, fuels breast cancer cell proliferation
Source: Cell Death Differ. 2020 Nov 23;28(5):1493–511. doi: 10.1038/s41418-020-00677-9 (PMC8166963; doi:10.1038/s41418-020-00677-9)

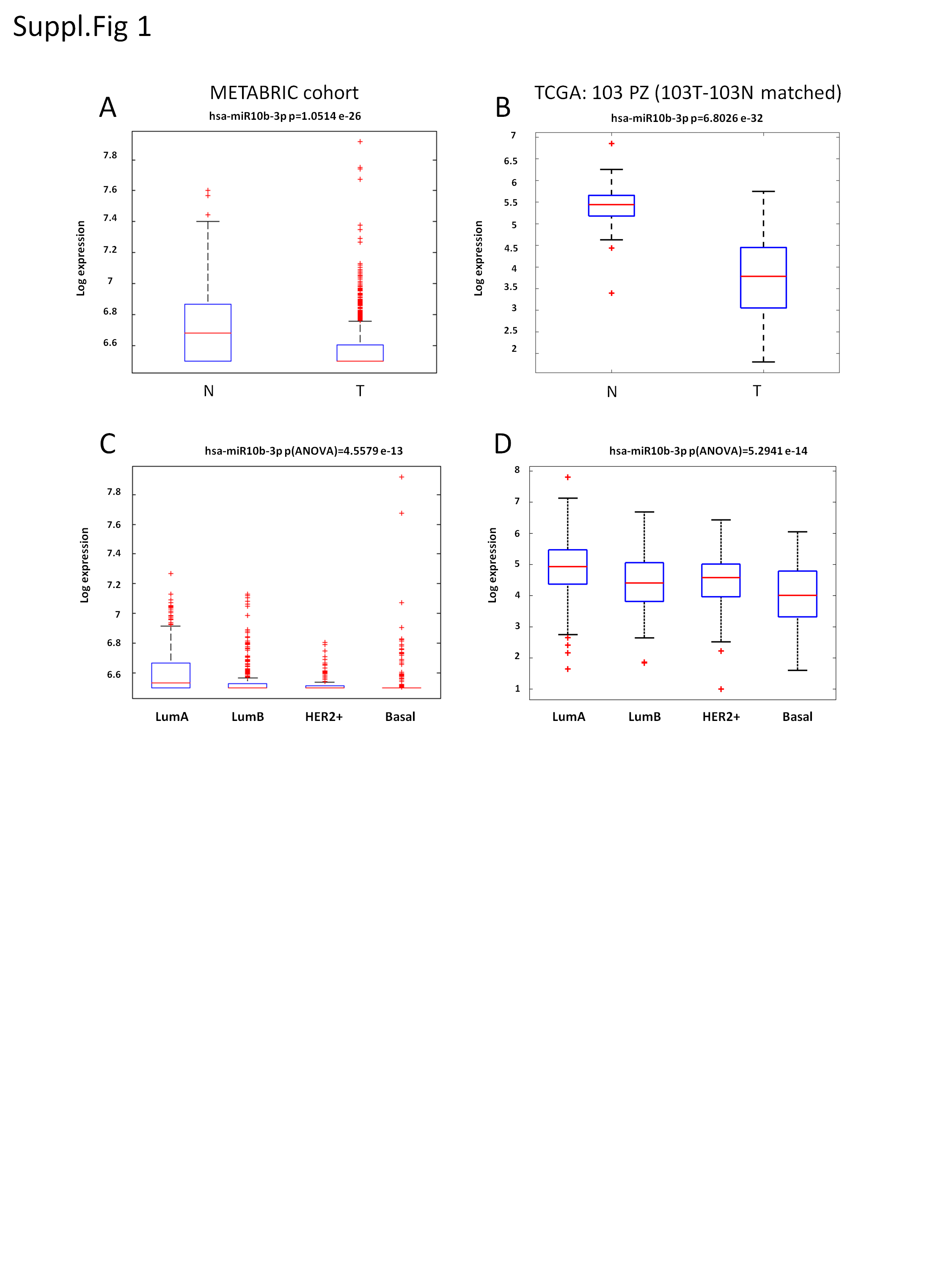

Supplement: Supplementary file 2 — Suppl. Fig1 [file 41418_2020_677_MOESM2_ESM.tif]

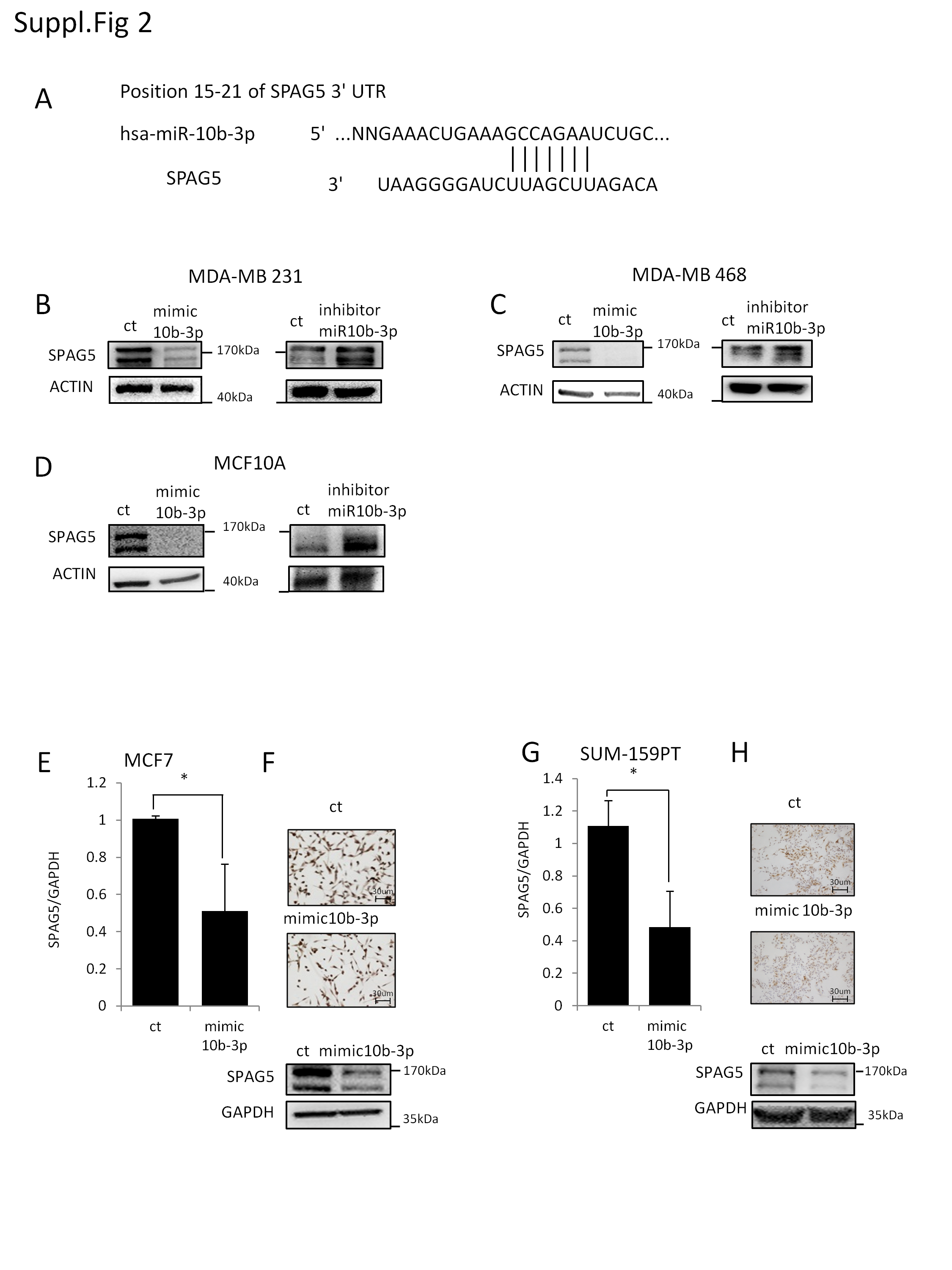

Supplement: Supplementary file 3 — Suppl. Fig 2 [file 41418_2020_677_MOESM3_ESM.tif]

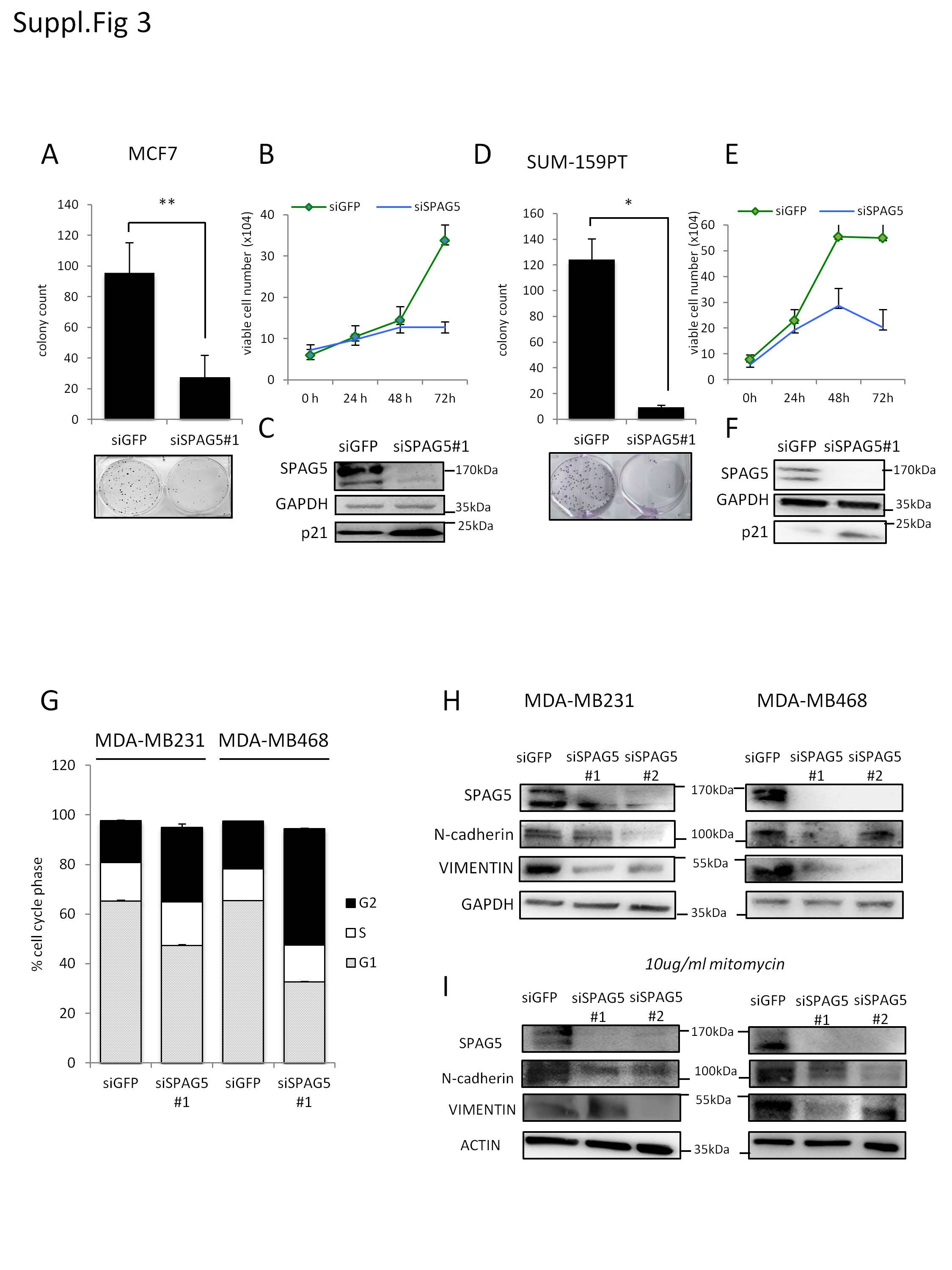

Supplement: Supplementary file 4 — Suppl. Fig 3 [file 41418_2020_677_MOESM4_ESM.tif]

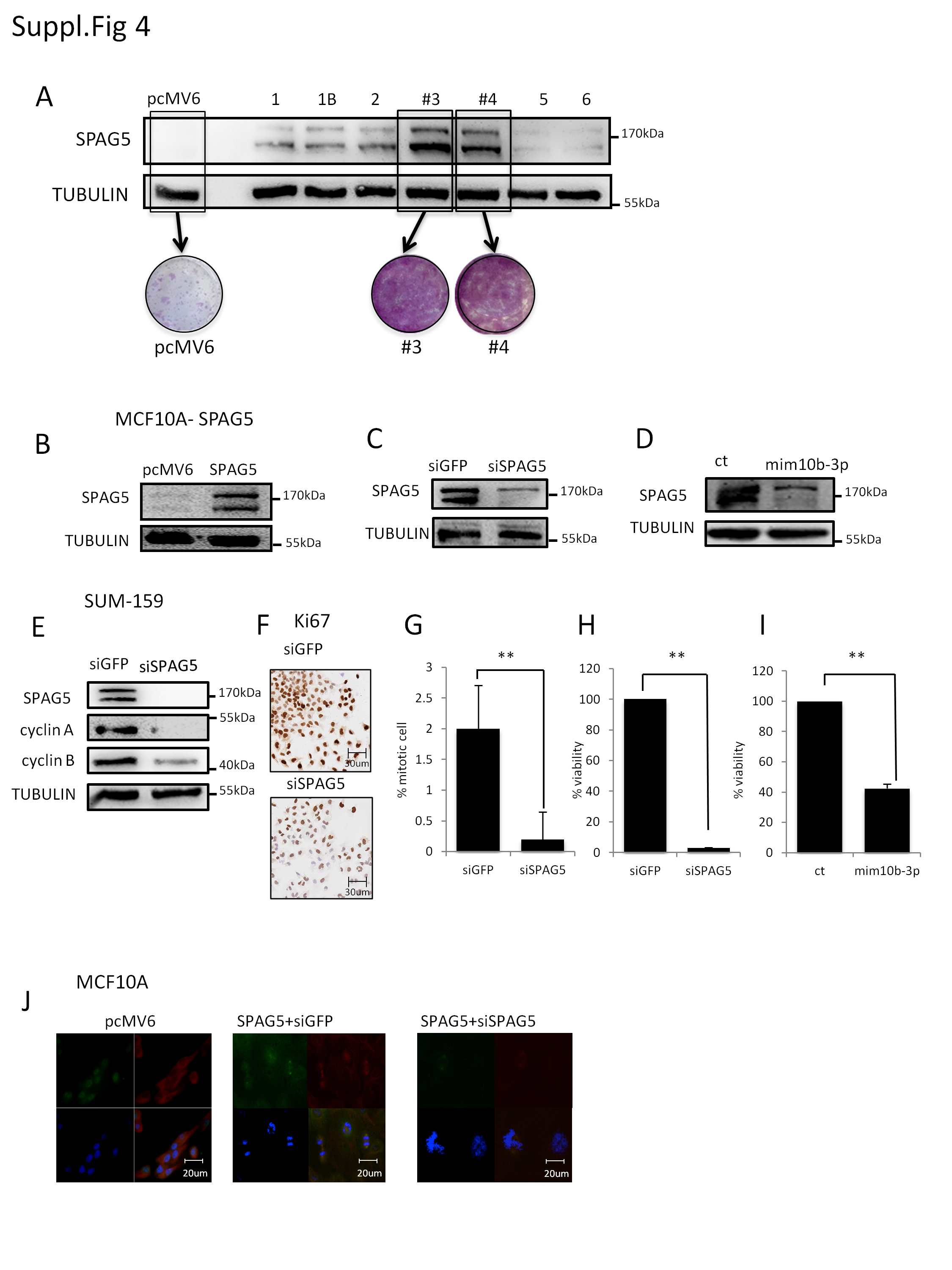

Supplement: Supplementary file 5 — Suppl. Fig 4 [file 41418_2020_677_MOESM5_ESM.tif]

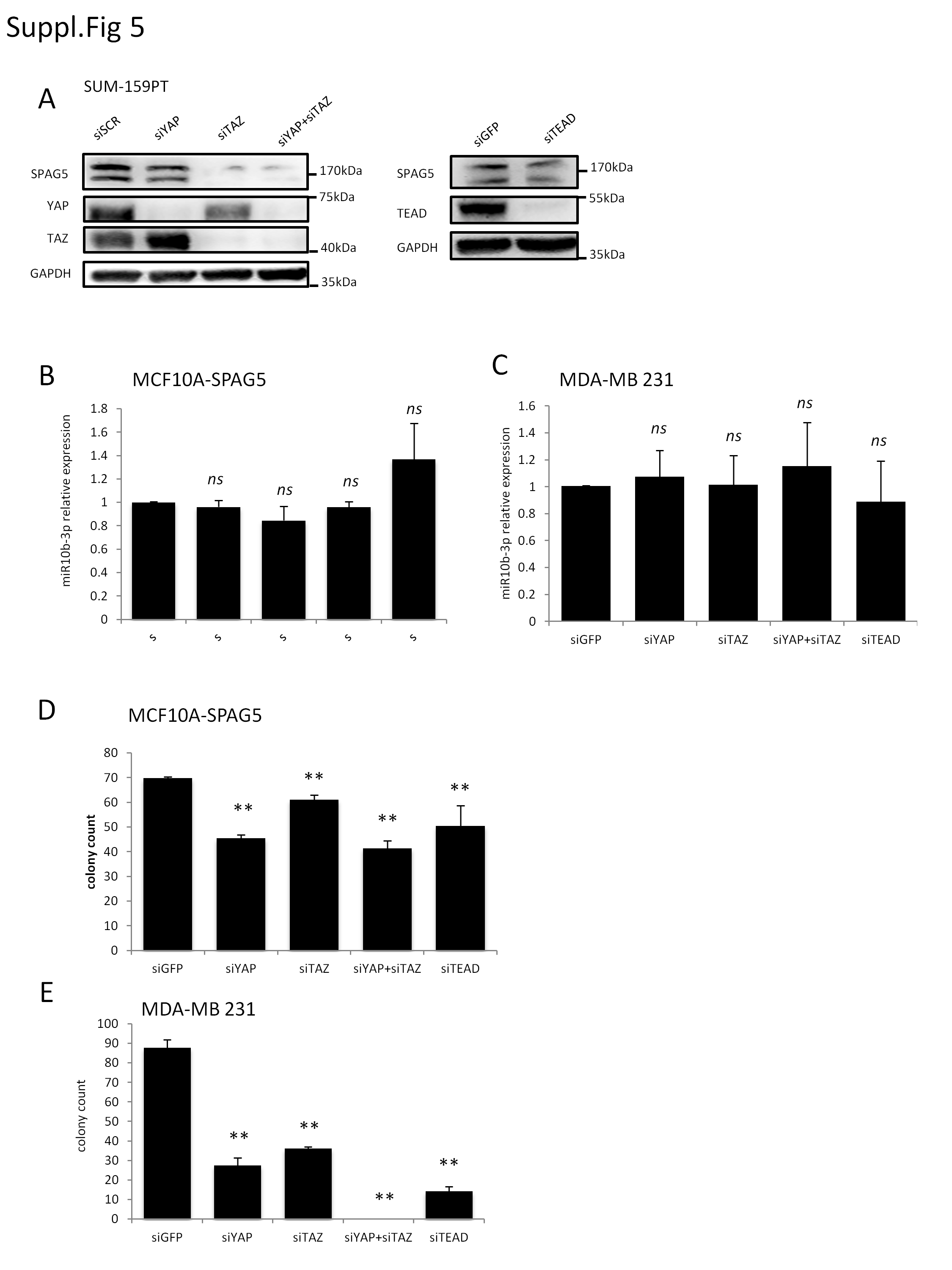

Supplement: Supplementary file 6 — Suppl. Fig 5 [file 41418_2020_677_MOESM6_ESM.tif]

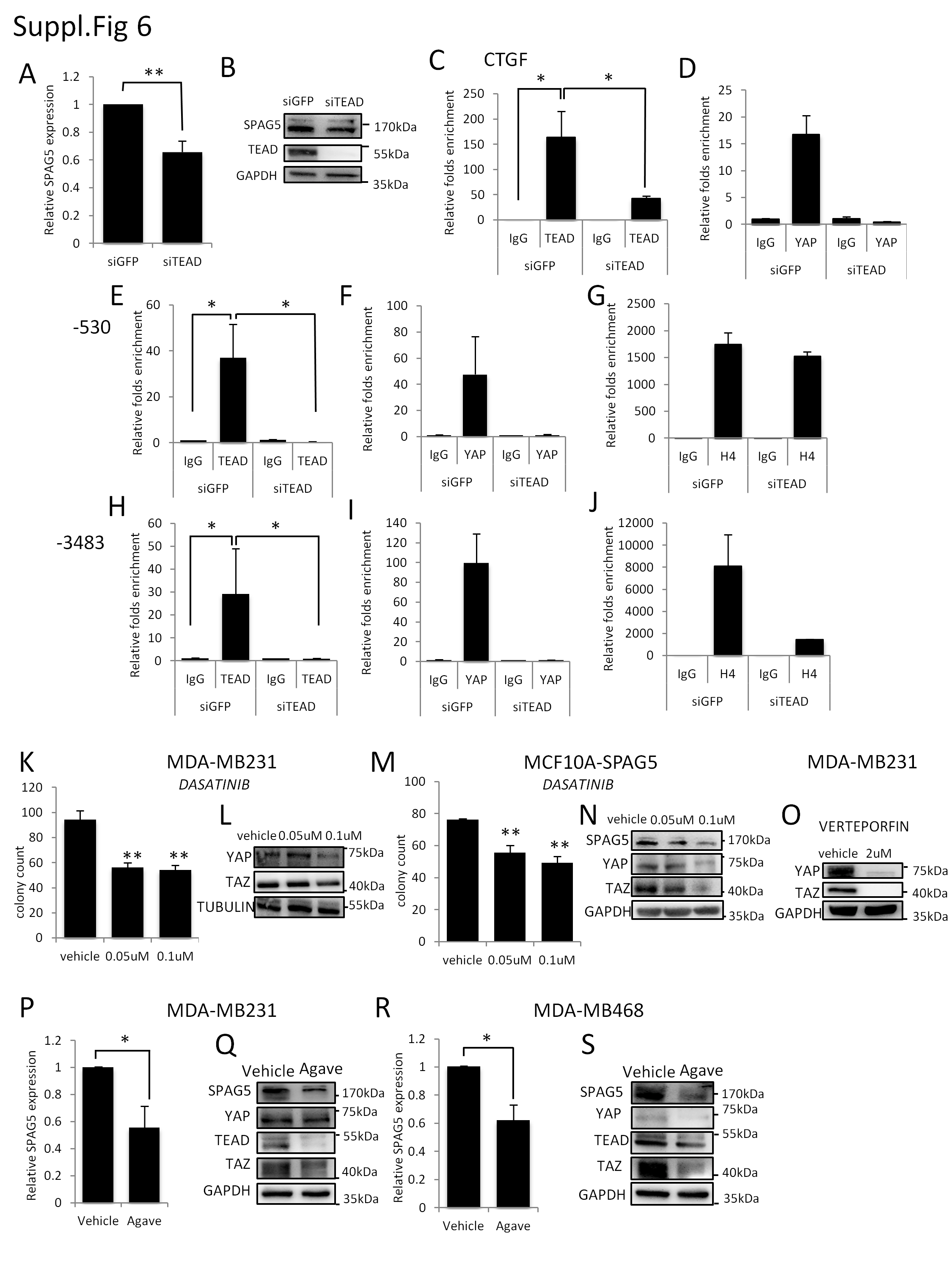

Supplement: Supplementary file 7 — Suppl. Fig 6 [file 41418_2020_677_MOESM7_ESM.tif]
